# Alkanediamide-Linked Bisbenzamidines Are Promising Antiparasitic Agents

**DOI:** 10.3390/ph9020020

**Published:** 2016-04-19

**Authors:** Jean J. Vanden Eynde, Annie Mayence, Madhusoodanan Mottamal, Cyrus J. Bacchi, Nigel Yarlett, Marcel Kaiser, Reto Brun, Tien L. Huang

**Affiliations:** 1 College of Pharmacy, Xavier University of Louisiana, New Orleans, LA 70125, USA; jean-jacques.vandeneynde@ex.umons.ac.be (J.J.V.E.); annie.mayence@condorcet.be (A.M.); 2 RCMI Cancer Research Center, Xavier University of Louisiana, New Orleans, LA 70125, USA; mmottama@xula.edu; 3 Department of Chemistry, Xavier University of Louisiana, New Orleans, LA 70125, USA; 4 Haskins Laboratories and Department of Biological and Health Sciences, Pace University, 1 Pace Plaza, New York, NY 10038, USA; cbacchi@pace.edu; 5 Haskins Laboratories and Department of Chemistry and Physical Sciences, Pace University, 1 Pace Plaza, New York, NY 10038, USA; nyarlett@pace.edu; 6 Swiss Tropical and Public Health Institute, Socinstrasse 57, Basel CH-4002, Switzerland; marcel.kaiser@unibas.ch (M.K.); reto.brun@unibas.ch (R.B.)

**Keywords:** antiparasitics, bisbenzamidines, DNA binding, *Plasmodium falciparum*, *Trypanosoma brucei*

## Abstract

A series of 15 alkanediamide-linked bisbenzamidines and related analogs was synthesized and tested *in vitro* against two *Trypanosoma brucei* (*T.b.*) subspecies: *T.b. brucei* and *T.b. rhodesiense*, *Trypanosoma cruzi*, *Leishmania donovani* and two *Plasmodium falciparum* subspecies: a chloroquine-sensitive strain (NF54) and a chloroquine-resistant strain (K1). The *in vitro* cytotoxicity was determined against rat myoblast cells (L6). Seven compounds (**5**, **6**, **10**, **11**, **12**, **14**, **15**) showed high potency against both strains of *T. brucei* and *P. falciparum* with the inhibitory concentrations for 50% (IC_50_) in the nanomolar range (IC_50_ = 1–96 nM). None of the tested derivatives was significantly active against *T. cruzi* or *L. donovani*. Three of the more potent compounds (**5**, **6**, **11**) were evaluated *in vivo* in mice infected with the drug-sensitive (Lab 110 EATRO and KETRI 2002) or drug-resistant (KETRI 2538 and KETRI 1992) clinical isolates of *T. brucei*. Compounds **5** and **6** were highly effective in curing mice infected with the drug-sensitive strains, including a drug-resistant strain KETRI 2538, but were ineffective against KETRI 1992. Thermal melting of DNA and molecular modeling studies indicate AT-rich DNA sequences as possible binding sites for these compounds. Several of the tested compounds are suitable leads for the development of improved antiparasitic agents.

## 1. Introduction

**Figure 1:**
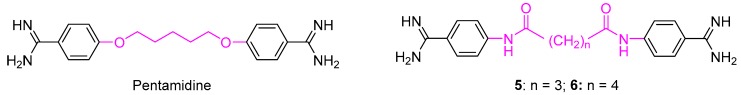
Chemical structures of pentamidine and lead compounds.

We previously reported on the promising anti-*Pneumocystis* activity of two key alkanediamide-linked bisbenzamidines when evaluated in an animal model of pneumocystosis [1,2]. Based on the results with the two lead compounds, the synthesis of an expanded series of alkanediamide-linked bisbenzamidines and the *in vitro* anti-*Pneumocystis* and anti-trypanosomal activities were reported [3]. The *in vitro* results showed excellent activity against drug-sensitive and -resistant isolates of *Trypanosoma brucei* with low cytotoxicity in the A549 human lung carcinoma cell line. The design strategy of replacing the strong electron-donating ether functions of the pentyldioxylinker in pentamidine with poor electron-donating amide functions in the lead compounds (**5** and **6**, Figure 1) resulted in highly potent antiprotozoal agents with greatly reduced cytotoxicity. In this study, we wish to report on the antiparasitic activity of the expanded series of alkanediamide-linked bisbenzamidines against several other parasitic protozoa such as *T. cruzi*, *L. donovani*, *P. falciparum* as well as their *in vivo* efficacy in several animal models of trypanosomiasis infected with drug-sensitive (Lab 110 EATRO and KETRI 2002) or -resistant (KETRI 2538 and 1992) clinical isolates [4] of *T.b. rhodesiense*.

There is a great need for new antiparasitic agents with improved efficacy and low toxicity. Current chemotherapy is inadequate for many parasitic diseases because of limited efficacy, unacceptable side effects, and increasing occurrence of resistance. Development of an effective vaccine for these parasitic diseases have proven ineffective, therefore, chemotherapy with existing drugs is the only available therapeutic option. Parasitic diseases infect almost one sixth of the world population (>one billion people), with malaria being the most common. Besides its antimicrobial properties, the bisbenzamidines class of agents, represented by pentamidine, has demonstrated a wide range of biological properties [5] including anticancer, antidiabetic, antiviral and antiparasitic properties. However, pentamidine is clinically used only in the treatment of *Pneumocystis jirovecii* pneumonia, early stage human African trypanosomiasis and antimony-resistant leishmaniasis. The development of a novel prodrug from the bisbenzamidine class, parafuramidine maleate (DB289) against HAT and *Pneumocystis* pneumonia, was halted in a phase III sleeping sickness clinical trial because of nephrotoxicity in healthy volunteers [6]. Despite this setback, there is considerable interest in developing novel and safer bisbenzamidines [7] and several of these compounds are in various stages of drug development [8].

## 2. Results and Discussion

### 2.1. Chemistry

**Scheme 1:**
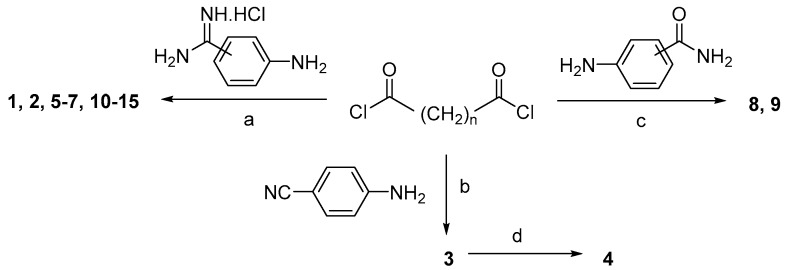
General procedures for the synthesis of compounds **1**–**15**. *Reagents and conditions*: (a) DMF, pyridine, reflux, 30 min–2 h; (b) and (c): Dioxane, room temp., stirred overnight; (d) Hydroxylamine, DMSO, 70 °C, 17 h.

The syntheses of the targeted compounds are illustrated in Scheme 1. It is to be noted that the complete synthetic and structural characterization have been described for compounds **3**–**6** and **8** in the literature [1,3,9] but not for the other compounds reported in this study. The appropriate diacid chlorides were reacted with aminobenzamidine or aminobenzonitrile or aminobenzamide to obtain the bisbenzamidines (**1**, **2**, **5**–**7**, **10**–**15**), bisbenzonitrile (**3**) and bisbenzamides (**8**, **9**) in fair yields. The bisbenzamidoxime **4** is readily obtained by reacting **3** with hydroxylamine. Compound **4** is specifically designed to function as a prodrug with improved oral bioavailability by masking the highly basic amidine functions with the less basic amidoxime moieties. We recently demonstrated that the amidoxime moiety in **4** is readily metabolized to the amidine function following incubation *in vitro* with mouse liver microsomes [9].

### 2.2. Pharmacology

The structure-activity relationship of 15 alkanediamide-linked bisbenzamidines (including analogs) and their antiparasitic activity were studied. Several of the bisbenzamidines were characterized with high potency against the two strains of *T. brucei* and *P. falciparum* (Table 1). Bisbenzamidines linked with the diamide moiety of the type Bza–NHCO–(CH_2_)_n_–CONH–Bza where Bza = benzamidine, *n* = 3, 4, 5, 6, 8, 10 showed high potency against both strains of *T. brucei* and *P. falciparum* in the low nanomolar range (IC_50_ = 1–96 nM) irrespective of the length of the methylene chain. Compound **1** was also highly active (IC_50_ = 3–8 nM) against both strains of *P. falciparum*, but was inactive against the other parasitic protozoa. None of the tested derivatives was active against *T. cruzi* or *L. donovani*. The tested compounds exhibited low cytotoxicity against the non-carcinoma L6 rat skeletal myoblasts cell line.

The selectivity index of these compounds for *T. brucei* (SI*_T.b_*) or *P. falciparum* (SI*_P.f_*) are defined as the ratio of the L6 cells IC_50_ to the *T. brucei* or *P. falciparum* IC_50_ values. Based on this definition, the most selective compounds in this series against *T. brucei* was compound **6** with SI > 50,000, whereas compounds **1** and **5** were the most selective against *P. falciparum* with SI > 33,000. The selectivity indices for the reference compounds, pentamidine or chloroquine, could not be calculated since they were not tested in the L6 cells. The remarkable low cytotoxicity and high selectivity of some of the compounds, especially **5** and **6** when compared with pentamidine, were demonstrated earlier with the lung A549 [3] lung NCI-H460, CNS SF-268 and breast MCF7 carcinoma cell lines [10]. *In vivo* evaluation in the immunosuppressed mouse model of *Pneumocystosis* demonstrated that compound **5** was highly efficacious against the infection at 20 mg/kg and 40 mg/kg, with >1000-fold reductions in burden, and resulted in improved survival curves *versus* those of pentamidine treated mice at the same doses [2]. The replacement of the dioxypentyl linker in pentamidine with the diamidealkyl linker resulted in compounds with reduced toxicity. This might be attributed to the lower lipophilicity (see Table 2) and different pathways of metabolism of these compounds since it has been suggested that the phenolic linkage in pentamidine might be involved in the toxicity of the compound [11]. Further characterization of the metabolites of our compounds would be needed to confirm this hypothesis.

Because of the promising *in vitro* activities, several compounds (**5**, **6**, **11**) were selected for *in vivo* efficacy studies using mice infected with drug-susceptible (Lab 110 EATRO and KETRI 2002) and drug-resistant (KETRI 2538 and 1992) strains of *T*. *brucei* (Table 3). The compounds were administered via the intraperitoneal route at doses ranging from 1.0 mg/kg/day to 25.0 mg/kg/day for 3 days unless otherwise noted. Pentamidine was used as the reference compound. All three of the tested compounds were effective in curing mice infected with the Lab 110 EATRO or KETRI 2002 strains at several doses. Compound **11** was the most effective against these two strains since it also cured the mice at lower doses (1.0 or 2.5 mg/kg). Two of the tested compounds, namely **5** and **6**, were highly efficacious in curing mice infected with the drug-resistant KETRI 2538 clinical isolate at several doses. However, the bisbenzamidines including pentamidine were ineffective in curing mice infected with the drug-resistant KETRI 1992 isolate, although the tested compound **5** prolonged the mean survival days of the treated animals to 25 days at the 25 mg/kg dose.

The *in vivo* results reported here as well as the earlier results with pneumocystosis [2] indicate that several of the bisbenzamidines reported here are suitable leads for further optimization to develop orally active prodrugs. These compounds generally differ from pentamidine in having lower lipophilicity, greater H-bond donor capability and polar surface area (Table 2). The bisamidine moieties have similar calculated basicity (pKa~10.68) when compared to pentamidine (pKa~10.94). Several of them have “lead-like” characteristics based on physicochemical properties. Therefore, modifying the leads into prodrugs with greater oral bioavailability would be a rational approach to develop more effective antiparasitic agents. In fact, we have already reported our initial attempts in this approach [9] by selecting compound **5** as a lead in which the amidine functions were masked with less basic and more lipophilic moieties that can readily be biotransformed into the active amidine functions. Further *in vitro* metabolism study of these potential prodrugs is in progress.

Bisbenzamidines such as pentamidine, propamidine and furamidine have been recognized as promising antimicrobial agents against bacteria, protozoa, fungi and amoeba [7]. This class of compounds has demonstrated excellent activity against *Pneumocystis, Trypanosoma* spp., *Leishmania* spp., and *Plasmodium* spp. [2,3,14,15]. These compounds have been proposed to act by initially binding to DNA at AT-rich sites followed by inhibition of one or more of several DNA dependent enzymes (e.g., topoisomerases and nucleases) or by direct inhibition of the transcription process [16,17]. In kinetoplastid parasites such as African trypanosomes, the bisbenzamidines are postulated to be taken up by transporters and concentrate in the mitochondrion leading to destruction of the kinetoplast DNA [7]. In the case of *P. falciparum*, the nuclear DNA has been implicated as the bioreceptor target [18]. However, other possible modes of action cannot be ruled out. These include the binding of these compounds to ferriprotoporphyrin IX and inhibiting the formation of hemozoin [15,19,20], disruption of polyamine metabolism [21], and interactions with a host of non-conventional biomolecular targets [5]. These observations suggest that dicationic diamidines are pleotropic compounds that are capable of interacting with multiple targets.

In addition to the determination of the potency of the synthesized compounds against different strains of cell lines (Table 1), thermal melting experiments of these compounds were also conducted with poly(dA-dT) and calf thymus DNA (Table S1, supplementary file). In general, the ΔT_m_ values are higher for poly(dA-dT) and a correlation was obtained between the ΔT_m_ of poly(dA-dT) and the pIC_50_ values of these compounds in *T.b. brucei* (*r* = 0.73), *T.b. rhodesiense* (*r* = 0.79) (see Figure S1A,B, supplementary file) and *P. falciparum* K1 (*r* = 0.67) (Figure S1C, supplementary file). The weaker binding to DNA (lower ΔT_m_ values) compared to pentamidine might be attributed to the lower lipophilicity and higher PSA properties of these compounds since they would be expected to bind less favorably in the less polar environment of the minor grove of DNA as was reported for the aza-analogues of furamidine [11]. Our results suggest that the antiparasitic activities of the benzamidine analogs against *T.b. brucei*, *T.b. rhodesiense* and *P. falciparum* K1 are associated with their ability to bind to AT rich oligomers, as reported in the literature [7,20,22]. However, it should be noted that compound **5** and several bisbenzamidines with various linkers were shown to interfere with hemozoin formation at micromolar concentrations in *ex vivo* experiments [15]. Therefore, ability of these compounds to act at other potential targets cannot be ruled out.

### 2.3. Molecular Modeling Studies

In order to examine how these compounds bind to the minor groove of AT-rich DNA duplex and their preferred affinities for specific AT-rich binding sites, we have done docking studies as described in the supplementary section. Docking scores and correlation coefficients (*r* and *p*) between the pIC_50_ and dockings scores are tabulated in Tables S1 and S2 (supplementary file). Correlation data indicates that our compounds have high preference for binding to the minor groove of a single G or GC inserted AT-rich DNA duplexes. In the active compound **15**, the terminal amidines displayed three H-bonds and the linker diamide formed one H-bond with the nucleotides of DNA. The detailed binding interactions of compounds **11** and **15** with the DNA can be found in the supplementary section (Figure S2). In the crystal structure of pentamidine in complex with AT-rich DNA, one of the terminal amidines formed one H-bond with N3 of A5 and a close contact to O4′ of A6, and the other terminal amidine formed one H-bond with N3 of A17, a close contact to O4′ of A18 and one water mediated contact to O4′ of G10 [23]. While it may not be a direct comparison, as in the crystal structure our model also exhibited at least two H-bond interactions between the terminal amidines and the nucleotide. Thus, the observed hydrogen bond interactions and the appropriate curvature of the compounds to match the convex shape of the DNA minor groove may be responsible for the higher affinity of active compounds. This study shows that our compounds better bind to G or GC inserted AT-rich sites. While these models provide a tool to study the trend in binding of our compounds to different oligomers, for an exact representation of the drug-DNA interactions X-ray or NMR structures are more desirable.

## 3. Experimental Section

### 3.1. Chemistry

^1^H-NMR spectra were obtained using a Varian Inova instrument (500 MHz) (Agilent Technologies, Santa Clara, CA, USA), and chemical shifts (δ) are given in ppm using TMS as internal reference and coupling constants (*J*) in Hz. IR spectra were recorded on a Perkin-Elmer Spectrum One spectrometer (PerkinElmer, Waltham, MA, USA) operating in the diffuse reflectance mode. High resolution mass spectra (HRMS) were recorded on an Orbitrap mass spectrometer (Thermo Fisher Scientific, Waltham, MA, USA) with electrospray ionization (ESI). Solvents and reagents were commercially available (Aldrich, St. Louis, MO, USA; Fisher Scientific, Pittsburg, PA, USA) and were used without further purification. Elemental analysis was carried out by M-H-W laboratories, Phoenix, AZ. Analysis of C, H, N were within ±0.4% of theoretical values. Compounds **3** [3], **4** [9], **5** [3], **6** [1] and **8** [3] have been described in the literature.

#### General Synthesis of Bisbenzamidines **1**, **2**, **7**, **10**–**15**

A mixture of 4-aminobenzamidine and/or 3-aminobenzamidine (10 mmol), pyridine (8 mL, 100 mmol), appropriate diacid chloride (5 mmol), in *N*,*N*-dimethyl formamide (40 mL) was refluxed for 30–120 min. The resultant precipitate was filtered and washed with the appropriate solvents.

*N,N*′*-bis[4-(aminoiminomethyl)phenyl] ethane-1,2-dicarboxamide* (**1**). The product was successively washed with water and acetone to yield 10% solid, m.p. > 300 °C; IR:3130, 1674, 1611, 1514, 1412 cm^−1^; ^1^H-NMR: (DMSO-d_6_) δ11.1 (s, br, 2H); 9.2 (s, br, 8H); 8.0 (d, 4H, *J* = 9 Hz), 7.9 (d, 4H, *J* = 9 Hz) ppm. Anal. Calcd. for C_16_H_16_N_6_O_2_·2HCl·1.5 H_2_O (424.28): C, 45.29; H, 4.98; N, 19.80. Found: C, 45.55; H, 5.12; N, 19.84.

*N,N*′*-bis[4-(aminoiminomethyl)phenyl] butane-1,4-dicarboxamide* (**2**). The product was successively washed with ethanol and ether to yield 40% solid, m.p. > 300 °C; IR:3030, 1669, 1602, 1541, 1415 cm^−1^; ^1^H-NMR:(DMSO-d_6_) δ10.5 (s, 2H); 9.2 (s, 4H); 8.8 (s, 4H); 7.8 (m, 8H); 2.7 (s, br, 4H) ppm. Anal. Calcd. for C_18_H_20_N_6_O_2_·2HCl (424.28): C, 50.83; H, 5.21; N, 19.76. Found: C, 50.77; H, 5.44; N, 19.58.

*N,N*′*-bis[3-(aminoiminomethyl)phenyl] hexane-1,6-dicarboxamide* (**7**). The product was washed twice with acetone to yield 57% solid, m.p. > 300 °C; IR:3054; 1668; 1564; 1482; 1411; 1314 cm^−1^; ^1^H-NMR:(DMSO-d_6_) δ10.5 (s, 2H); 9.3 (s, 4H); 9.1 (s, 4H); 8.1 (s, 2H); 7.8 (d, 2H); 7.5 (t, 2H); 7.3 (d, 2H); 2.4 (t br, 4H); 1.7 (t br, 4H) ppm. Anal. Calcd. for C_20_H_24_N_6_O_2_·2HCl (453.37): C, 52.98; H, 5.78; N, 18.54. Found: C, 52.74; H, 5.99; N, 18.45.

*N-[3-(aminoiminomethyl)phenyl] N*′*-[4-(aminoimino methyl)phenyl] hexane-1,6-dicarboxamide* (**10**). Oily liquid, % yield unknown. IR:3086; 1667; 1601; 1481; 1315; 1264 cm^−1^; ^1^H-NMR:(DMSO-d_6_) δ10.7 (d, 1H); 10.5 (d, 1H); 9.4 (s, 2H); 9.2 (s, 2H); 9.1 (s, 4H); 9.0 (s, 4H); 8.2 (s, 1H); 7.8 (m, 5H); 7.5 (t, 1H); 7.4 (d, 1H); 2.4 (m br, 4H); 1.6 (m br, 4H) ppm. HRMS: found 381.2057, calcd. 381.2039 for C_20_H_24_N_6_O_2_.

*N,N*′*-bis[4-(aminoiminomethyl)phenyl] heptane-1,7-dicarboxamide* (**11**). The product was successively washed with ethanol and ether to yield 45% solid, m.p. > 300 °C; IR:3117, 1675, 1616, 1541, 1496 cm^−1^; ^1^H-NMR:(DMSO-d_6_) δ10.5 (s, 2H); 9.2 (s, 4H); 8.9 (s, 4H); 7.8 (m, 8H); 2.3 (t, 4H, *J* = 7 Hz); 1.6 (q, 4H, *J* = 7 Hz); 1.3 (q, 2H, *J* = 7 Hz) ppm. Anal. Calcd. for C_21_H_30_N_6_O_2_·2HCl.0.2H_2_O) (503.42): C, 50.10; H, 6.40; N, 16.69. Found: C, 50.38; H, 6.03; N, 16.51.

*N,N*′*-bis[4-(aminoiminomethyl)phenyl] octane-1,8-dicarboxamide* (**12**). The product was successively washed with water, ethanol and ether to yield 10% solid, m.p. > 300 °C; IR:3063, 1674, 1609, 1541, 1338, 1308 cm^−1^; ^1^H-NMR:(DMSO-d_6_) δ10.5 (s, 2H); 9.2 (s, 4H); 8.9 (s, 4H); 7.8 (m, 8H); 2.3 (t, 4H, *J* = 7 Hz); 1.6 (m, br, 4H); 1.3 (m, br, 4H) ppm. Anal. Calcd. for C_22_H_28_N_6_O_2_·2HCl) (481.42): C, 54.89; H, 6.28; N, 17.46. Found: C, 55.12; H, 6.25; N, 17.28.

*N,N′-bis[4-(aminoiminomethyl)phenyl] nonane-1,9-dicarboxamide* (**13**). After refluxing for 2 h, the cooled solution was poured into acetone (100 mL). The product was successively washed with acetone and water to yield 50% solid, m.p. > 300 °C; IR:2935, 1696, 1674, 1541, 1330 cm^−1^; ^1^H-NMR:(DMSO-d_6_) δ10.4 (s, 2H); 9.3 (s, br, 8H); 7.8 (m, 8H); 2.3 (t, 4H, *J* = 7 Hz); 1.6 (q, br, 4H); 1.3 (q, br, 6H, *J* = 7 Hz) ppm. Anal. Calcd. for C_23_H_32_N_6_O_2_·2HCl·0.5C_2_H_5_OH) (518.48): C, 55.60; H, 6.80; N, 16.21. Found: C, 55.98; H, 6.61; N, 15.96.

*N,N′-bis[4-(aminoiminomethyl)phenyl] decane-1,10-dicarboxamide* (**14**). After refluxing for 2 h, thecooled solution was poured into acetone (100 mL). The product was successively washed with acetone and water to yield 50% solid, m.p. > 265 °C decomposed; IR:2931, 1667, 1599, 1559, 1487 cm^−1^; ^1^H-NMR:(DMSO-d_6_) δ 10.4 (s, 2H); 9.1 (s, br, 8H); 7.8 (m, 8H); 2.4 (t, 4H, *J* = 7 Hz); 1.6 (br m, 4H); 1.3 (br m, 8H, *J* = 7 Hz) ppm. HRMS: found 437.2672, calcd. 437.2665 for C_24_H_32_N_6_O_2_.

*N,N′-bis[4-(aminoiminomethyl)phenyl] dodecane-1,12-dicarboxamide* (**15**). The product was successively washed with water, ethanol and ether to yield 35% solid, m.p. > 300 °C; IR:3093, 1669, 1602, 1541, 1411 cm^−1^; ^1^H-NMR:(DMSO-d_6_) δ 10.4 (s, 2H); 9.2 (s, 4H); 8.9 (s, 4H); 7.8 (m, 8H); 2.3 (t, 4H, *J* = 8 Hz); 1.6 (q, br, 4H); 1.3 (m, br, 12H) ppm. HRMS: found 465.2985, calcd. 465.2978 for C_26_H_36_N_6_O_2_.

### 3.2. Biology

#### 3.2.1. *In Vitro* Antiparasitic Activity

The two clinical isolates of *T. brucei* used for the *in vitro* study were *T. b. brucei* Lab 110 EATRO (pentamidine-sensitive) and *T. b. rhodesiense* KETRi 243 (melarsoprol, pentamidine- and diminazene diaceturate-resistant) [14]. The *in vitro* assays with the two isolates of *T. brucei* were conducted according to previously described procedures [14]. The anti-*T. cruzi* activity was determined using intracellular amastigote forms of *T. cruzi* Tulahuen strain C2C4 in rat skeletal myoblasts (L6 cells) as described previously [24]. The anti-*L. donovani* activity was determined using the amastigotes of *L. donovani* strain MHOM-ET-67/L82 as described previously [24]. The anti-plasmodial activity against the two strains of *P. falciparum* NF54 (chloroquine-sensitive) and K1 (chloroquine-resistant) were determined using the erythrocytic stage of *P. falciparum* as described previously [24]. The cytotoxicity assays against rat skeletal myoblast L6-cells were performed as described previously [24].

#### 3.2.2. *In Vivo* Anti-Trypanosomal Activity in Mice

The *in vivo* studies were conducted according to the procedures described previously [4,14].

#### 3.2.3. DNA Binding Affinity Measurements

The DNA binding affinity of the tested compounds was determined by measuring the change in mid-point of the thermal denaturation curves for a 1:5 compound to DNA base pair ratio as described previously [15]. Each T_m_ value reported in Table S1 (supplementary file) represents the mean of at least two experimental determinations.

### 3.3. Molecular Modeling Studies

We used Surflex-Dock to study the DNA binding affinities of 16 bisbenzamidine compounds to the minor groove of AT-rich sites and an AT absent site. Computational details are described in supplementary section.

## 4. Conclusions

This study reports on the synthesis of a series of alkanediamide-linked bisbenzamidines (and analogs) and their *in vitro* and *in vivo* evaluation as potential antiparasitic agents. Potential cytotoxicity of the compounds in a mammalian cell line, as well as their interactions with DNA via thermal denaturation and molecular modeling studies, have been determined. Seven of the compounds (**5**, **6**, **10**, **11**, **12**, **14**, **15**) were highly potent (IC_50_ = 1–96 nM) against drug-sensitive as well as drug-resistant cell lines of *T. brucei* and *P. falciparum*. Three compounds (**5**, **6**, **11**) were highly effective in curing mice infected with the drug-sensitive strains (Lab 110 EATRO and KETRI 2002) of *T. brucei*, whereas **5** and **6** were also efficacious in curing mice infected with the drug-resistant KETRI 2538 clinical isolate. Thermal denaturation studies with DNA showed that these compounds have stronger binding to poly(dA-dT) *versus* calf thymus DNA and a good correlation was observed between the ΔT_m_ of poly(dA-dT) and the antiparasitic activity of these compounds against *T. brucei* and *P. falciparum*. Molecular modeling studies indicated that these compounds exhibited high preference for binding to the minor groove of a single G or GC inserted AT-rich (AAAGTTT or AAAGCTTT) DNA duplexes and they correlated well with the pIC_50_ values of the compounds against *T. brucei* and *P. falciparum*.

## Figures and Tables

**Table 1:** *In vitro* antiparasitic and cytotoxic properties of alkanediamide-linked bisbenzamidines and analogs.

Compd.	n	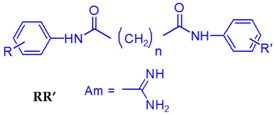		IC_50_ (µM) ^a^						
				*T.b. brucei* ^b^	*T.b. rhod* ^c^	*T. cruzi*	*L. donovani*	*P. falcip*NF54 ^d^	*P. falcip*K1 ^e^	L6 Cells
**1**	1	p-Am	p-Am	ND	ND	>100	>100	**0.008**	**0.003**	>100
**2**	2	p-Am	p-Am	9.0	2.19	>100	>100	ND	0.29	28.0
**3**	3	p-CN	p-CN	6.50	7.90	>100	>100	ND	44.8	>100
**4**	3	p-C(=NOH)NH_2_	p-C(=NOH)NH_2_	7.30	10.0	>100	>100	ND	3.16	>100
**5**	3	p-Am	p-Am	**0.009**	**0.096**	>100	>100	**0.002**	**0.002**	69.0
**6**	4	p-Am	p-Am	**0.003**	**0.002**	>100	>100	**0.004**	**0.018**	>100
**7**	4	m-Am	m-Am	0.041	0.021	>100	>100	ND	0.38	>100
**8**	4	p-CONH_2_	p-CONH_2_	2.80	1.10	>100	>100	ND	>100	>100
**9**	4	m-CONH_2_	m-CONH_2_	NT	1.97	>100	>100	ND	>100	46.4
**10**	4	p-Am	m-Am	**0.012**	**0.007**	>100	>100	**0.015**	**0.004**	>100
**11**	5	p-Am	p-Am	**0.002**	**0.002**	>100	>100	**0.002**	**0.002**	43.0
**12**	6	p-Am	p-Am	**0.003**	**0.001**	>100	>100	**0.002**	**0.006**	38.4
**13**	7	p-Am	p-Am	0.400	0.240	99.5	67.4	**0.002**	**0.008**	49.2
**14**	8	p-Am	p-Am	**0.002**	**0.004**	76.4	68.5	**0.014**	**0.012**	78.6
**15**	10	p-Am	p-Am	**0.008**	**0.007**	80.9	10.7	**0.032**	**0.015**	80.2
Ref				0.002 ^f^	0.002 ^f^	1.13 ^g^	0.44 ^h^	0.006 ^i^	0.18 ^i^	0.010 ^j^

^a^ Each value is the average of at least two determinations. ^b,c^ The *Trypanosoma brucei brucei* strain was Lab 110 and the *Trypanosoma brucei rhodesiense* strain was KETRI 243. Data for both strains are obtained from [3]; ^d,e^ The *Plasmodium falciparum* strains NF 54 and K1 are chloroquine-sensitive and -resistant strains respectively; ^f^ pentamidine; ^g^ benznidazole; ^h^ miltefosine; ^i^ chloroquine; ^j^ podophyllotoxin. ND denotes not done.

**Table 2:** Calculated physicochemical properties, Lipinski rule of 5 and lead likeness.

Compd.	MolWt	LogP	^a^ H-don Count	^b^ H-acc Count	^c^ Ring #	LogD	^d^ Rot Bonds	^e^ PSA	Lipinki Rule of 5	Lead Likeness
**1**	338.37	0.38	6	6	2	−4.44	6	157.94	3 of 4	4 of 6
**2**	352.40	0.31	6	6	2	−4.5	7	157.94	3 of 4	4 of 6
**3**	332.36	2.63	2	4	2	2.63	6	105.78	4 of 4	6 of 6
**4**	398.42	0.75	6	8	2	0.74	8	175.42	3 of 4	5 of 6
**5**	366.42	0.76	6	6	2	−4.06	8	157.94	3 of 4	4 of 6
**6**	380.45	1.20	6	6	2	−3.61	9	157.94	3 of 4	5 of 6
**7**	380.45	1.20	6	6	2	−3.6	9	157.94	3 of 4	5 of 6
**8**	382.42	1.06	4	6	2	1.06	9	144.38	4 of 4	6 of 6
**9**	382.42	1.06	4	4	2	1.06	9	144.38	4 of 4	6 of 6
**10**	380.45	1.20	6	6	2	−3.61	9	157.94	3 of 4	5 of 6
**11**	394.48	1.64	6	6	2	−3.17	10	157.94	3 of 4	5 of 6
**12**	408.50	2.09	6	6	2	−2.72	11	157.94	3 of 4	4 of 6
**13**	422.53	2.53	6	6	2	−2.28	12	157.94	3 of 4	4 of 6
**14**	436.56	2.98	6	6	2	−1.84	13	157.94	3 of 4	4 of 6
**15**	466.61	3.87	6	6	2	−0.95	15	157.94	3 of 4	4 of 6
**16 ^f^**	340.42	2.32	4	6	2	−2.50	10	118.20	4 of 4	6 of 6

All the values reported in this Table were obtained using the Marvin Suite of ChemAxon [12] and the pKa values were obtained using the Schrodinger Suite [13]. Lipinski Rule of 5 (MolWt ≤ 500, LogP ≤ 5, ^a^ Number of H-donor atoms ≤ 5, ^b^ Number of H-acceptor atoms ≤ 10). Lead Likeness (MolWt ≤ 450, LogD (on pH 7.4) ≥ −4 and ≤ 4, Number of H-donor atoms ≤ 5, Number of H-acceptor atoms ≤ 8, ^c^ Number of rings ≤ 4, ^d^ Number of rotatable bonds ≤ 10,). ^e^ Polar Surface Area, ^f^ Pentamidine.

**Table 3:** *In vivo* trypanocidal activity of selected compounds in mice ^a^.

Clinical Isolate	Compd.	Dosage (mg/kg/Day)	Mean Survival (Days)	No. of Mice Cured/Total (%)
Lab 110 EATRO ^b^	None	-	5.0	0/3
	Pentamidine	1.0, 2.5, 5, 10	>30	5/5 (100) ^c^
	**5**	1.0	6.0	0/3
		2.5	11.3	0/3
		5	10	2/3 (66)
		10	>30	3/3 (100)
		15	>30	3/3 (100)
	**6**	1.0	6.7	0/3
		2.5	10.0	0/3
		5	>30	3/3 (100)
		10	>30	3/3 (100)
		15	>30	3/3 (100)
	**11**	1.0, 2.5, 5, 10	>30	3/3 (100) ^c^
KETRI 2002 ^b^	None	-	9.0	0/3
	Pentamidine	1.0, 5, 10	>30	5/5 (100) ^c^
	**5**	10, 15, 25	>30	3/3 (100) ^c^
	**6**	10, 15, 25	>30	3/3 (100) ^c^
	**11**	1.0, 2.5, 5, 10	>30	3/3 (100) ^c^
KETRI 2538 ^b^	None	-	4.3	0/3
	Pentamidine	1.0, 5, 10	>30	5/5 (100) ^c^
	**5**	10, 15, 25	>30	3/3 (100) ^c^
	**6**	5, 10, 15	>30	3/3 (100) ^c^
KETRI 1992 ^b^	None	-	7.4	0/5
	Pentamidine	1	12.6	0/3
		5	17.0	0/3
		10	22.2	0/3
	**5**	10	17.5	0/3
		15	14.5	0/3
		25	25	0/3

^a^ *In vivo* efficacy of compounds given via i.p route *vs.* several clinical isolates of *T. brucei*. Mice were infected with 250,000 parasites and dosing commenced 24 h post infection. Mice were separated into groups of three and injected i.p once a day for 3 days unless otherwise noted. Infected untreated controls were used for each experiment. Mice were considered cured if surviving more than 30 days beyond death of the last control without parasites in tail vein blood smears. Mean survival (in days) of animals dying of trypanosomiasis is exclusive of cured animals. ^b^ Trypanosome strains. *T.b. brucei* Lab 110 EATRO strain is susceptible to standard trypanocides including the diamidines. The following are clinical isolates of *T.b. rhodesiense*: KETRI 243, 2002, 2538 and 1992. Strain 2002 is susceptible to standard trypanocides including the diamidines. Strains refractory to DFMO are KETRI 243 and 2538. Strains refractory to arsenical drugs are KETRI 243, 1992 and 2538. Strains refractory to diamidines are KETRI 243 and 1992 (see [4] for details). ^c^ All doses cured. Groups of 3 or 5 animals used for all doses.

## Supplementary Materials

The following are available online at http://www.mdpi.com/1424-8247/9/2/20/s1, Figure S1: Correlation between the experimental pIC_50_ in different cell lines and the ΔT_m_ with poly(dA-dT), Figure S2: Detailed views from the minor groove of the hydrogen bond interactions between compounds **11** and **15** and the central –AAAGTTT- sites of 5’-d(CCAAAGTTTGC)-3’ duplex, Table S1: Docking of alkanediamide-linked bisbenzamidines and analogs to various DNA duplexes with specific central sequences, *in vitro* antiparasitic properties (pIC_50_) and thermal melting data (ΔT_m_) of tested analogs with poly(dA-dT) and CT-DNA, Table S2: Pearson’s correlation (*r*) between the experimental pIC_50_ values of three cell lines or ΔT_m_ of poly(dA-dT) and the docking scores for different central minor groove of sequences. Details of molecular modeling studies.
