# Supplementary material for: Alkanediamide-Linked Bisbenzamidines Are Promising Antiparasitic Agents
Source: Pharmaceuticals (Basel). 2016 Apr 19;9(2):20. doi: 10.3390/ph9020020 (PMC4932538; doi:10.3390/ph9020020)
Supplement: Supplementary file 1 [file pharmaceuticals-09-00020-s001.pdf]

Supplementary Materials: Alkanediamide-Linked Bisbenzamidines Are Promising Antiparasitic Agents

Jean J. Vanden Eynde , Annie Mayence , Madhusoodanan Mottamal, Cyrus J. Bacchi, Nigel Yarlett , Marcel Kaiser, Reto Brun and Tien L. Huang

1. Thermal Melting Data, pIC_{50} Data and Docking Scores

Table S1. Docking of alkanediamide-linked bisbenzamidines and analogs to various DNA duplexes with specific central sequences, *in vitro* antiparasitic properties (pIC_{50}) and thermal melting data (ΔTm) of tested analogs with Poly(dA-dT) and CT-DNA.

Compd.	Central DNA Sequences of the Duplex and the Docking Scores				pIC_{50} Values for 3 Cell Lines			Thermal Melting	
	AAA TTT	AAA GTTT	AAAG CTTT	GCG CGC	*T.b. brucei*	*T.b. rhod* [a]	*P. falcip* K1 [b]	ΔTm, °C Poly(dA-dT)	ΔTm, °C CT-DNA [c]
**1**	4.71	4.79	5.74	6.29	ND	ND	8.523	ND	ND
**2**	6.26	6.01	7.16	5.23	5.046	5.660	6.538	11.5	8.1
**3**	5.88	5.95	5.03	6,32	5.187	5.102	4.349	0.15	−0.2
**4**	6.82	5.37	7.16	7.22	5.137	5.000	5.500	0.0	0.7
**5**	6.48	6.62	6.94	6.31	8.046	7.018	8.699	11.4	5.6
**6**	7.28	7.79	7.92	7.04	8.523	8.699	7.745	14.4	8.7
**7**	7.01	8.46	6.98	6.61	7.387	7.678	6.420	13.8	ND
**8**	1.99	5.09	5.01	5.39	5.553	5.959	4.000	0.0	ND
**9**	6.45	6.12	6.08	5.96	ND	5.706	4.000	0.0	ND
**10**	7.12	7.59	8.07	7.03	7.921	8.155	8.398	ND	ND
**11**	6.85	8.91	8.54	4.63	8.699	8.699	8.699	10.7	6.4
**12**	6.49	8.42	8.35	7.15	8.523	9.000	8.222	13.2	7.1
**13**	9.19	9.3	8.79	8.35	6.398	6.620	8.097	7.5	4.6
**14**	8.68	10.53	11.1	9.01	8.699	8.398	7.921	10.0	6.6
**15**	9.57	10.06	8.99	6.42	8.097	8.155	7.824	8.5	ND
**16**[d]	6.99	8.83	8.64	6.26	8.699	8.699	6.745	21.6	11.6

[a] *T.b. rhodesiense*. [b] *P. falciparum* K1; [c] Calf Thymus DNA; [d] Pentamidine; ND denotes not done.

2. Correlation Plot

Correlation between the experimental pIC_{50} for *T.b. brucei*, *T.b. rhodesiense* and *P. falciparum* K1 cell lines and the ΔT_m with poly(dA-dT) are shown in Figure S1.

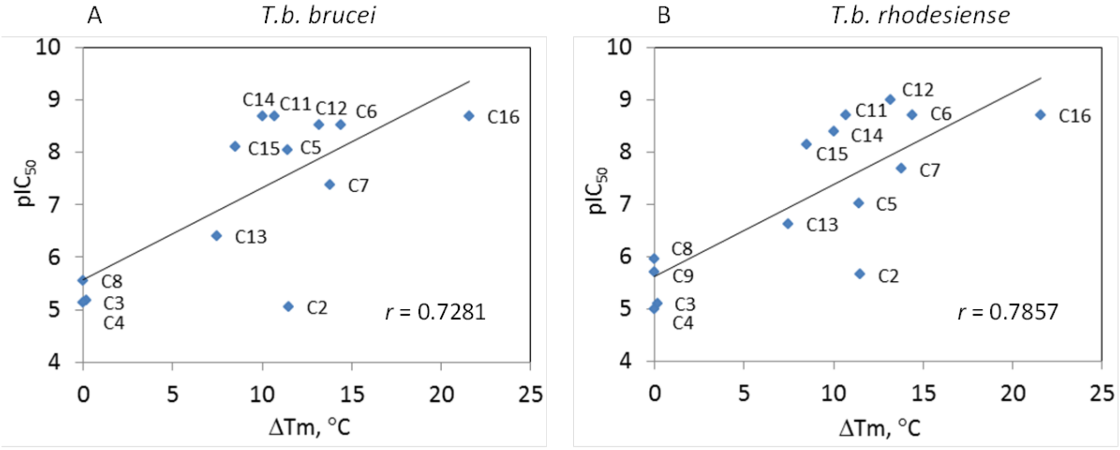

Figure S1. *Cont.*

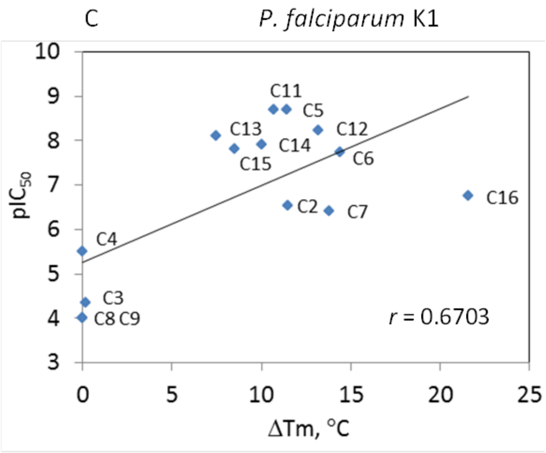

Figure S1. Correlation between the experimental pIC_{50} in different cell lines and the ΔT_m with poly(dA-dT). (**A**) *T.b. brucei*, (**B**) *T.b. rhodesiense* and (**C**) *P. falciparum* K1 cell lines. The *p* values for the correlations are 0.005, 0.001 and 0.009 for *T.b. brucei*, *T.b. rhodesiense* and *P. falciparum* K1 respectively.

3. Molecular Modeling Studies

3.1. Methodology

We used Surflex-Dock to study the DNA binding affinities of 16 bisbenzamidine compounds to the minor groove of AT-rich sites. The AAATTT sequence was the primary target of interest, but we also considered to target AAAGTTT and AAAGCTTT, because a single G or GC inserted short AT base pairs are common in eukaryotic parasite genomes. One DNA sequence rich in G-C base pairs (5′-d(GCGCGCGCGC)-3′) was also considered. For the AAATTT sequence we used 5′-d(CCAAATTTGC)-3′ as used in a recent study [2]. The AAAGTTT and AAAGCTTT sequences were constructed by inserting G and GC to the center of the 5′-d(CCAAATTTGC)-3′ sequence. The initial regular B-DNA duplexes were constructed using the Biopolymer/Build module in SYBYL-X1.3 and energy minimized using the Powell method and Tripos force filed. Initial 3D structures of all the l6 compounds were built using the sketch molecule function in SYBYL-X and energy minimized using the Powell method with Tripos force field and Gasteiger-Huckel charges.

To help position the bound bisbenzamidine compounds to the minor groove constituting AT rich base pairs, the model d(CCAAATTTGC) structure was aligned to the crystal structure of d(CGCGAATTCGCG)-pentamidine complex (ID64.pdb) [1] such that the central AATT sites were overlaid and the DNA sequence from 1D64 was removed. All the other model DNA duplexes were then aligned to this model such that crystal ligand is in the minor groove of the target sequences AAATTT, AAAGTTT, AAAGCTTT and GCGCGC. All the docking calculations were carried out using the standard protocols with Surflex-Dock, and 20 poses were retained for each compound based on the docking scores. Within the top 10 poses at least 4 to 9 poses were found to have identical or very similar poses. The standard deviations of the energies of these poses for most compounds were less than 0.5. A consensus score that combines multiple popular scoring functions, such as G_Score, PMF_Score, D_Score and ChemScore, was also calculated for each hit, and it is supposed to be more robust than any single function for evaluating ligand-receptor interaction and it has been validated in virtual high throughput screening [3]. Thus the total score with the best consensus score was used for ranking the binding affinities of benzamidine compounds to the double helix DNA with specific AT rich and GC sites.

3.2. Surflex-Dock Data

Docking scores of alkanediamide-linked bisbenzamidines and analogs to various DNA duplexes with specific central sequences are tabulated in Table S1.

3.3. Correlation Data

Correlation between the experiment pIC_{50} values in different cell lines and the docking score haven determined. The Pearson *r* values and the *p* values for statistical significance are tabulated in Table S2.

Table S2. Pearson's correlation (*r*) between the experimental pIC_{50} values of three cell lines or ΔTm of poly(dA-dT) and the docking scores for different central minor groove sequences. The values in brackets are the *p* values.

**Cell Lines or Polymer**	**Correlation Coefficient *r* and the *p* Values between the Docking Scores and the pIC_{50} or ΔTm.**			
	**AAATTT**	**AAAGTTT**	**AAAGCTTT**	**GCGCGC**
T.b. brucei	0.43 (0.127)	0.75 (0.002)	0.68 (0.008)	0.19 (0.526)
T. b. rhod	0.39 (0.150)	0.78 (0.001)	0.70 (0.004)	0.18 (0.519)
P. falcip K1	0.48 (0.060)	0.51 (0.042)	0.62 (0.010)	0.26 (0.321)
Poly(dA-dT)	0.35 (0.218)	0.58 (0.028)	0.56 (0.038)	0.06 (0.832)

3.4. DNA-Ligand Binding Interactions

Two active compounds, **11** and **15**, bound to the central –AAAGTTT- sites of 5′-d(CCAAAGTTTGC)-3′ duplex are depicted in Figure S2. In compound **11** one terminal amidine displayed a H-bond with O3′ of A5 and the other terminal amidine displayed a H-bond with O3′ of C17. In compound **15** one terminal amidine displayed two H-bonds with O4′ of A5 and N3 of A4, and the other terminal amidine displayed a H-bond with O3′ of A16. In addition, the alkanediamide nitrogens of the linkers in **11** formed two H-bonds with O2 of T7 and N3 of A5 and in **15** formed one H-bond with O2 of T8.

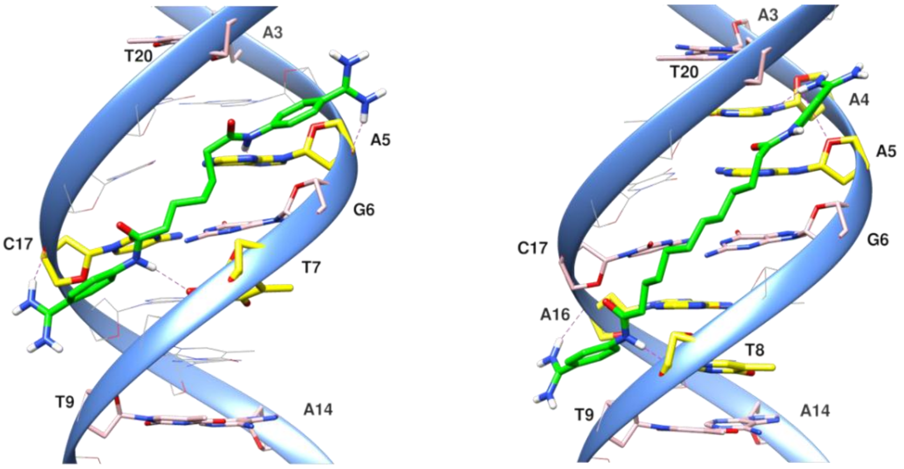

(A) Compound **11** (B) Compound **15**

Figure S2. Detailed views from the minor groove of the hydrogen bond interactions between compounds **11** (**A**) and **15** (**B**) and the central –AAAGTTT- sites of 5′-d(CCAAAGTTTGC)-3′ duplex. The far end AT base pairs, the center GC base pair and the nucleotides that make hydrogen bonds with the ligands are shown in stick model. H-bond making nucleotides are shown with yellow carbons and other nucleotides are shown in pink carbons. The DNA is represented as blue ribbon. Al the oxygen and nitrogen atoms are colored in red and blue respectively, and the carbon atoms of the compounds are shown in green.

References

1. Edwards, K.J.; Jenkins, T.C.; Neidle, S. Crystal structure of a pentamidine-oligonucleotide complex: Implications for DNA-binding properties. *Biochemistry* **1992**, *31*, 7104–7109.

2. Chai, Y.; Paul, A.; Rettig, M.; Wilson, W.D.; Boykin, D.W. Design and synthesis of heterocyclic cations for specific DNA recognition: From at-rich to mixed-base-pair DNA sequences. *J. Org. Chem.* **2014**, *79*, 852–866.
3. Clark, R.D.; Strizhev, A.; Leonard, J.M.; Blake, J.F.; Matthew, J.B. Consensus scoring for ligand/protein interactions. *J. Mol. Graph. Model.* **2002**, *20*, 281–295.

© 2016 by the authors; licensee MDPI, Basel, Switzerland. This article is an open access article distributed under the terms and conditions of the Creative Commons by Attribution (CC-BY) license (http://creativecommons.org/licenses/by/4.0/).
